# Effect of differentiated direct‐to‐pharmacy PrEP refill visits supported with client HIV self‐testing on clinic visit time and early PrEP continuation

**DOI:** 10.1002/jia2.26222

**Published:** 2024-03-06

**Authors:** Kidist Belay Zewdie, Kenneth Ngure, Margaret Mwangi, Dominic Mwangi, Simon Maina, Lydia Etyang, Gakuo Maina, Vallery Ogello, Emmah Owidi, Nelly R. Mugo, Jared M. Baeten, Kenneth K. Mugwanya

**Affiliations:** ^1^ Department of Epidemiology University of Washington Seattle Washington USA; ^2^ Department of Global Health University of Washington Seattle Washington USA; ^3^ Department of Community Health Jomo Kenyatta University of Agriculture and Technology Nairobi Kenya; ^4^ Partners in Health Research and Development Center for Clinical Research, Kenya Medical Research Institute Nairobi Kenya; ^5^ Department of Medicine University of Washington Seattle Washington USA

**Keywords:** PrEP, direct‐to‐pharmacy, HIV prevention, differentiated care delivery, clinic wait time, Kenya

## Abstract

**Introduction:**

Delivery of oral pre‐exposure prophylaxis (PrEP) is being scaled up in Africa, but clinic‐level barriers including lengthy clinic visits may threaten client continuation on PrEP.

**Methods:**

Between January 2020 and January 2022, we conducted a quasi‐experimental evaluation of differentiated direct‐to‐pharmacy PrEP refill visits at four public health HIV clinics in Kenya. Two clinics implemented the intervention package, which included direct‐to‐pharmacy for PrEP refill, client HIV self‐testing (HIVST), client navigator, and pharmacist‐led rapid risk assessment and dispensing. Two other clinics with comparable size and client volume served as contemporaneous controls with the usual clinic flow. PrEP continuation was evaluated by visit attendance and pharmacy refill records, and time and motion studies were conducted to determine time spent in the clinics. Dried blood spots were collected to test for tenofovir‐diphosphate (TFV‐DP) at random visits. We used logistic regression to assess the intervention effect on PrEP continuation and the Wilcoxon rank sum test to assess the effect on clinic time.

**Results:**

Overall, 746 clients were enrolled, 366 at control clinics (76 during pre‐implementation and 290 during implementation phase), and 380 at direct‐to‐pharmacy clinics (116 during pre‐implementation and 264 during implementation phase). Prior to implementation, the intervention and control clinics were comparable on client characteristics (female: 51% vs. 47%; median age: 33 vs. 33 years) and PrEP continuation (35% vs. 37% at 1 month, and 37% vs. 39% at 3 months). The intervention reduced total time spent at the clinic by 35% (median of 51 minutes at control vs. 33 minutes at intervention clinics; *p*<0.001), while time spent on HIV testing (20 vs. 20 minutes; *p* = 0.50) and pharmacy (8 vs. 8 minutes; *p* = 0.8) was unchanged. PrEP continuation was higher at intervention versus the control clinics: 45% versus 33% at month 1, 34% versus 25% at month 3 and 23% versus 16% at month 6. TFV‐DP was detected in 85% (61/72) of samples, similar by the study group (83% vs. 85%).

**Conclusions:**

A client‐centred PrEP delivery approach with direct‐to‐pharmacy PrEP refill visits plus client HIVST significantly reduced clinic visit time by more than one‐third and improved PrEP continuation in public health HIV clinics in Kenya.

## 1 INTRODUCTION

Oral pre‐exposure prophylaxis (PrEP) is a highly effective HIV prevention strategy [1, 2, 3]; however, maximizing access and continuation is a key challenge for optimizing the overall public health impact of oral PrEP. In Kenya, PrEP is largely delivered through established public health clinics at no cost [4] but sustaining these programmes requires making its delivery more convenient for users while reducing the burden on the healthcare system. Major barriers to clinic‐based PrEP delivery include long waiting times, stigma, lack of privacy, time away from work and frequent clinic visits for refills [5, 6, 7, 8, 9, 10]. For HIV treatment programmes in Africa, patient‐centred models known as differentiated care service delivery tailor services to the client's clinical status and needs. These models have been shown to improve antiretroviral therapy (ART) adherence and viral suppression [11, 12, 13, 14]. Currently, community‐based models of differentiated PrEP delivery, such as community pharmacies and mobile clinics, are being tested [15, 16, 17, 18]. However, the infrastructure to support PrEP services in many African settings largely relies on fixed, government facilities for medication storage, staffing, prescribing, drug accountability, clinical oversight and management of side effects. These facilities are likely to continue playing a vital role even with the introduction of newer PrEP modalities. Therefore, various stakeholders, including the Kenya Ministry of Health (MOH), have advocated for the establishment of efficient facility‐based PrEP delivery that seamlessly integrates with existing services to ensure long‐term sustainability [19].

In public healthcare facilities across Kenya, PrEP services are delivered sequentially by different providers at different delivery points requiring clients to move between various stop points and often experience frequent queues along the way. For instance, HIV testing services (HTS) are provided by HTS providers at testing points followed by an adherence assessment by a counsellor, a clinical review by a clinician and dispensing by the pharmacy staff. One promising option to improve the efficiency and client‐centredness of facility‐based PrEP delivery is differentiated facility‐based pharmacy PrEP refill visits, where clients returning for refills can go straight to the pharmacy, skipping all other stop points. In addition, innovative and empowering technology such as HIV self‐testing (HIVST) can further enhance facility‐based PrEP delivery [20, 21, 22]. It is possible to optimize HIV testing by using innovative technology such as HIVST, which clients can perform while waiting for other services, enhancing efficiency while still upholding the core components of PrEP provision. However, there are limited data on the utility of differentiated facility pharmacy‐based PrEP provision, acceptability and safety of HIVST in the context of facility‐based PrEP delivery. In this prospective pilot implementation study, we evaluated the feasibility and efficiency of client‐centred direct‐to‐pharmacy PrEP refill visits supported with client HIVST to understand the effect on clinic time and PrEP continuation in African health facilities.

## 2 METHODS

### 2.1 Study setting and population

The study was conducted between September 2020 and January 2022 at four public health HIV clinics with established national PrEP programmes in the Central and Nairobi regions of Kenya. The four clinics previously participated in the Partners Scale‐Up Project [23]. The clinics were purposively selected based on the volume of clients accessing PrEP, space availability and willingness to implement the direct‐to‐pharmacy PrEP refill pathway. We enrolled clients aged ≥18 years who were receiving PrEP as part of the Kenya National PrEP programme at each of the four participating clinics [24].

### 2.2 Study design and implementation approach

This was a quasi‐experimental study of differentiated direct‐to‐pharmacy PrEP refill visits supported with client HIVST. Of the four clinics that expressed interest in implementing, two clinics implemented the intervention of direct‐to‐pharmacy PrEP care, while two comparable clinics served as contemporaneous controls with the usual clinic flow. Clinics were first matched 1:1 on the urban‐semi‐urban status and clinics in the resulting matched pair were then conveniently assigned to the intervention group. The study was executed in two phases: (1) a pre‐implementation phase (January 2020−August 2020) during which all four participating clinics implemented the usual clinic flow to document baseline clinic flow, PrEP initiation and continuation; and (2) an implementation phase (September 2020−January 2022), which began after two the intervention clinics started implementing the direct‐to‐pharmacy pathway. Importantly, during the implementation phase, each clinic implemented only one delivery pathway to eliminate the risk of provider and client confusion regarding delivery models.

### 2.3 Description of the intervention

The intervention package included: a PrEP client navigator to retrieve client files and direct clients to the pharmacy, client HIVST while waiting for refills, and direct‐to‐pharmacy refill visits with pharmacist‐led rapid risk assessment and dispensing. The implementation strategies to promote the direct‐to‐pharmacy model included training of existing healthcare providers and monthly debriefing meetings to provide feedback on clinic‐level PrEP initiation and continuation data. The intervention was differentiated for refill visits only but at the PrEP initiation visit at the intervention clinics, clients were informed that at their next return visit, they would receive PrEP services directly at the pharmacy and that they would have an opportunity to conduct HIVST. Clients also received OraQuick® HIVST (OraSure Technologies, USA) or Sure Check® (Chembio Diagnostic Systems, USA) kits and a brief demonstration on how to use them along with pictorial instructions translated into the local language, to take home for practice.

During the direct‐to‐pharmacy intervention, clients were dispensed PrEP quarterly, and at their return visits, they were directed to the facility pharmacy by the client navigator. While waiting at the pharmacy, clients were provided with HIVST kits to self‐test in a designated private space. Any positive results were required to be confirmed by a provider‐conducted standard blood rapid HIV test. After HIVST, clients presented their results to the pharmacy staff for cross‐validation. At the pharmacy, the pharmacy staff assessed for ongoing HIV risk, adherence, side effects, and acute HIV symptoms and dispensed PrEP. Throughout the intervention, clients had the option to seek help with the self‐testing process or see a clinical provider at any time if they had a medical indication or if they requested it. Because HIVST for PrEP services is not yet recommended by WHO due to concerns about slightly lower sensitivity relative to blood rapid diagnostic kits, PrEP users in the self‐testing intervention had standard provider‐conducted rapid HIV test at 6 months on PrEP to help quantify this potential risk [25].

### 2.4 Procedures for PrEP provision and commodity supply

At all clinics, PrEP delivery services were fully performed by existing MOH healthcare providers. PrEP eligibility was determined by self‐report based on characteristics associated with elevated risk for HIV (File S1) according to the Kenya MOH PrEP guidelines [25]. HIV testing kits and PrEP medication were supplied by the Kenya Medical Supplies Authority and provided for free to clients as part of the standard of care. The study project supported the provision of HIVST kits when the clinic ran out of supply. PrEP medication was prescribed for daily use by health providers at each of the facilities as per the Kenyan PrEP guidelines [23].

### 2.5 Outcome measures and data collection

For this implementation study, programme data were documented by existing facility staff on the standard MOH clinical encounter form, which is used for documenting individual‐level clinical records of clients on PrEP. During the study period, trained project‐dedicated staff abstracted client records including demographics, HIV behavioural factors, PrEP eligibility, and PrEP prescription and dispensing. In addition, we administered a brief structured survey to collect data on client experiences and satisfaction with HIVST, direct‐to‐pharmacy and waiting time. At each clinic, we conducted time and motion studies by following PrEP clients through the clinic from point of entry to exit to document time spent at each service point. Service and client‐level outcomes were evaluated at baseline, 1, 3 and 6 months. We collected dried blood spots (DBS) from clients returning for PrEP at randomly selected weeks to test for tenofovir‐diphosphate (TFV‐DP) levels. Quantitative TFV‐DP concentrations in DBS were determined by liquid chromatography‐tandem mass spectrometry at the University of Colorado.

### 2.6 Power and statistical analysis

We estimated that we would have >80% power to demonstrate at least a 30% reduction in total time spent at the clinic for PrEP services during the direct‐to‐pharmacy intervention versus usual care without adversely affecting early PrEP continuation (month 1). We conservatively assumed that about 40−50% of clients would refill at 1 month post‐initiation in the usual care based on the Partners Scale‐up project in a similar setting and population [26]. Thus, we estimated that 500 clients starting PrEP overall would provide sufficient power to show that at least 40% refilling at 1 month in the direct‐to‐pharmacy intervention would not be programmatically significantly worse than the usual care in the Partners Scale‐Up prior to the COVID‐19 pandemic.

The predictor of interest was the intervention status of the clinic at PrEP initiation. Only individuals who initiated PrEP during the implementation phase contributed to the primary evaluation of the intervention effect. Clients with prior PrEP use before the intervention contributed to the evaluation of clinic experience and satisfaction. The primary outcomes were time spent in a clinic by the client and continuation on PrEP at months 1 and 3 as secondary outcomes. Time was summarized as the total time spent at the clinic and separately as wait time and direct contact time with the provider at each delivery point. PrEP continuation was assessed by visit attendance and pharmacy PrEP refill records.

Descriptive statistics are summarized by the implementation phase and intervention group using median with interquartile range (IQR) for continuous variables and frequencies and proportions for categorical variables. We used generalized linear regression models with a logit link and robust standard errors to account for clustering by clinic, to assess the effect of the intervention on PrEP continuation. Covariates were included in multivariable models based on our knowledge of key drivers of PrEP use in this setting and included sex at birth, age, marital status, condom use and partner(s) HIV status. Wilcoxon rank‐sum test was used to assess the intervention effect on clinic time, separately for total clinic time, time for HIV testing and time at the pharmacy. All analyses were conducted in R software version 4.2 [27].

### 2.7 Ethical considerations

The study was reviewed and approved by the University of Washington Human Subjects Ethical Committee and the Scientific and Ethics Review Unit of the Kenya Medical Research Institute and participants provided written informed consent before data collection.

## 3 RESULTS

### 3.1 PrEP initiation and participant characteristics

Overall, 746 participants were enrolled (Table 1), 366 at the control clinics and 380 at the intervention clinics. Of the 366 participants who received PrEP at the control clinics, 76 initiated PrEP during the pre‐implementation phase, 47% were female, the majority (57%) were >30 years old and most participants (87%) reported having a partner living with HIV. At the intervention clinics, of the 380 participants who received PrEP, 116 initiated during the pre‐implementation phase: 51% were female, 59% were >30 years old, 81% were married or cohabiting and 64% reported having a partner living with HIV.

**Table 1:** General characteristics of study participants by implementation phase and intervention group

	Pre‐implementation phase		Implementation phase	
Characteristic	Control *n* = 76	Pre‐intervention *n* = 116	Control *n* = 290	Intervention^a^ *n* = 264
Sex				
Female	36 (47%)	59 (51%)	179 (62%)	149 (56%)
Male	40 (53%)	57 (49%)	111 (38%)	115 (44%)
Age (years)				
18−24	11 (14%)	20 (17%)	59 (20%)	41 (16%)
25−30	22 (29%)	28 (24%)	69 (24%)	65 (25%)
>30	43 (57%)	68 (59%)	162 (56%)	158 (60%)
Marital status				
Married/cohabiting	76 (100%)	94 (81%)	253 (87%)	202 (77%)
Single	0 (0%)	22 (19%)	37 (13%)	62 (23%)
HIV risk characteristic				
Has partner(s) living with HIV	66 (87%)	74 (64%)	141 (49%)	154 (58%)
High‐risk partner(s) with unknown HIV status	11 (14%)	35 (30%)	120 (41%)	100 (38%)
Multiple sex partners	2 (2.6%)	16 (14%)	30 (10%)	25 (9.5%)
Inconsistent or no condom use	21 (28%)	12 (10%)	82 (28%)	34 (13%)
Partner living with HIV is on ART	29 (58%)	47 (78%)	58 (73%)	119 (90%)

^a^ Intervention: Direct‐to‐pharmacy refill with client HIV self‐testing.

During the implementation phase, 290 clients initiated PrEP at the control clinics compared to 264 at the clinics implementing the direct‐to‐pharmacy intervention. Of 290 clients initiated on PrEP during the implementation phase at the control clinics, 62% were female, 56% were above the age of 30 and 49% reported having a partner living with HIV. Comparatively, among 264 clients at the direct‐to‐pharmacy intervention clinics, 56% were female, 60% were above the age of 30 and 58% reported having a partner living with HIV.

### 3.2 PrEP continuation and adherence to PrEP

Overall, 192 clients initiated PrEP during the pre‐implementation phase: 76 at the control clinics versus 116 at the two pilot clinics. During the pre‐implementation phase, the continuation of PrEP was statistically similar between the control and intervention clinics. Specifically, at the control clinics, 37%, 37% and 25% of clients continued PrEP at months 1, 3 and 6, respectively, compared to 35%, 39% and 26% of clients who continued PrEP at months 1, 3 and 6 (*p* >0.05 for all comparisons), respectively, at the intervention clinics.

During the implementation phase, a total of 554 clients initiated PrEP: 290 clients at the control clinics versus 264 at the direct‐to‐pharmacy intervention clinics. The intervention improved continuation on PrEP through 6 months compared to control clinics (Table 2). Specifically, continuation was 45% versus 33% at month 1 (adjusted odds ratio [adjOR] 1.51, 95% CI 1.04, 2.18, *p* = 0.03), 34% versus 25% at 3 months (adjOR 1.45, 95% CI 0.98, 2.14, *p* = 0.06) and 23% versus 16% at month 6 (adjOR 1.58 [1.01, 2.49], *p* = 0.047). Key correlates of PrEP continuation included age, marital status and reported partner's HIV status, with having a partner living with HIV demonstrating the strongest magnitude of association (Table 3). Specifically, clients with a partner living with HIV were over two‐fold more likely to continue PrEP at month 1 (adjOR 2.76, 95% CI 1.74−4.43, *p*<0.001), month 3 (adjOR 2.35, 95% CI 1.45−3.87, *p*<0.001) and month 6 (adjOR 3.66, 2.03, 6.79, *p*<0.001) compared to those reporting not to have a partner living with HIV.

**Table 2:** PrEP continuation and correlates of continuation by intervention type and month since initiation in the implementation phase (*n* = 554)

	Month 1			Month 3			Month 6		
Characteristic	PrEP continuation	AdjOR (95% CI)	*p*‐value	PrEP continuation	Adj OR (95% CI)	*p*‐value	PrEP continuation	Adj OR (95% CI)	*p*‐value
Intervention									
Direct‐to‐pharmacy	45% (118/264)	1.51 (1.04, 2.18)	0.03	34% (91/264)	1.45 (0.98, 2.14)	0.06	23% (61/264)	1.58 (1.01, 2.49)	0.047
Control clinics	33% (95/290)	Ref.		25% (73/290)	Ref.		16% (47/290)	Ref.	
Sex									
Male	45% (101/226)	1.03 (0.69, 1.53)	0.9	32% (72/226)	0.81 (0.53, 1.23)	0.3	23% (51/226)	0.78 (0.48, 1.26)	0.3
Female	34% (112/328)	Ref.		28% (92/328	Ref.		18 % (58/328)	Ref.	
Marital status									
Single	38% (38/99)	1.83 (1.06, 3.17)	0.032	27% (27/99)	1.43 (0.80, 2.56)	0.2	13% (13/99)	1.06 (0.49, 2.19)	0.9
Married/cohabiting	38% (175/455)	Ref.		30% (137/455)	Ref.		21% (96/455)	Ref.	
Age (years)	35 (27,44)	1.02 (1.01, 1.04)	0.009	33 (26,41)	1.02 (1.01, 1.04)	0.02	35 (27, 44)	1.02 (1.00, 1.04)	0.06
Partner(s) living with HIV									
Yes	47% (139/293)	2.76 (1.74, 4.43)	0.001	36% (106/293)	2.35 (1.45, 3.87)	<0.001	27% (80/293)	3.66 (2.03, 6.79)	<0.001
No	28% (71/258)	Ref.		22% (56/258)	Ref.		11% (28/258)	Ref.	
Consistent condom use									
No	32% (37/116)	1.04 (0.64, 1.67)	0.9	23% (27/116)	0.95 (0.56, 1.58)	0.8	19% (22/116)	1.68 (0.93, 2.98)	0.08
Yes	40% (173/435)	Ref.		31% (135/435)	Ref.		20% (86/435)	Ref.	

**Table 3:** Time spent in the clinic and on specific services (minutes) by the intervention group

Characteristic^a^	*N*	Direct‐to‐pharmacy with client HIVST, *n* = 58^b^	Usual clinic flow, *n* = 22^c^	*p*‐value^d^
Total clinic time	80	33.5 (29, 45)	50.5 (39, 104)	<0.001
Total time at the pharmacy	78	8 (6, 12)	8 (4, 15)	0.8
Direct contact time with pharmacy staff	78	7 (6, 8)	4 (3, 7)	0.001
Overall time for HIV testing	80	21 (18, 29)	25 (20, 42)	0.18
Direct time spent conducting HIV testing	80	20 (18, 27)	20 (18, 23)	0.46

^a^ Overall time includes time for the activity/services plus wait time, while direct time excludes wait for the respective activity/service.

^b and c^ Median (IQR).

^d^ Wilcoxon rank sum test.

**Figure 1:**
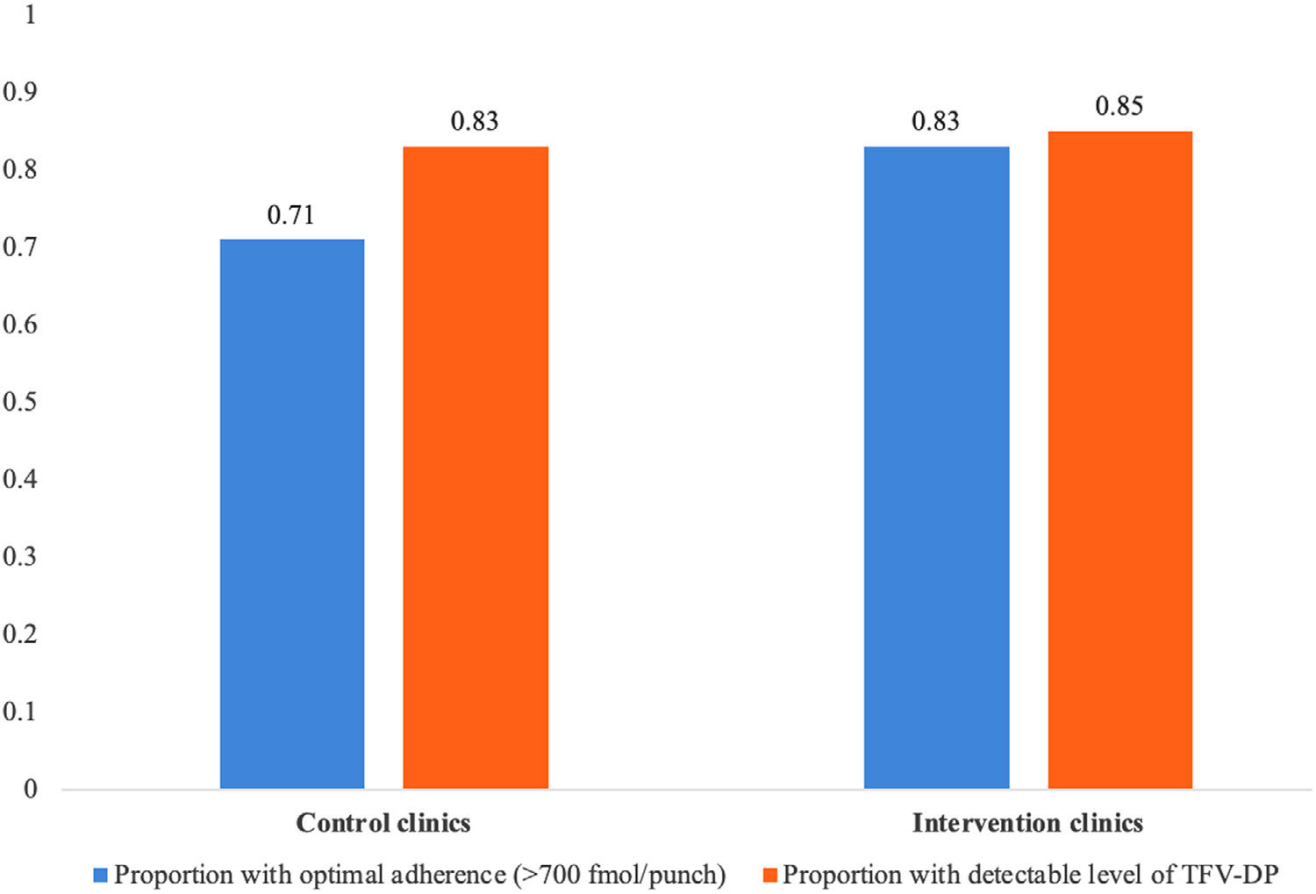
Drug detection as an objective measure of PrEP adherence at random visits in the control compared to intervention clinics (*n* = 71 samples).

A total of 72 DBS samples were collected at random visits from clients returning for PrEP refills. Overall, adherence was high in this population with 85% (61/72) of tested DBS samples indicating detectable TDF‐DP concentrations, similar in control versus intervention clinics (83% vs. 85%; Figure 1) Among DBS samples with quantifiable concentrations (*n* = 61), median (IQR) TFV‐DP concentrations was 1438 fmol/punch (978−1880), indicative of consistent daily dosing based on established benchmarks [28, 29]; 71% in control clinics and 85% in intervention clinics had concentrations indicative of consistent high adherence (>700 fmol/punch).

### 3.3 Effect of direct‐to‐pharmacy intervention on time spent in a clinic

A total of 80 participants were observed during the time and motion studies, 58 at the direct‐to‐pharmacy intervention and 22 at the control clinics. Overall, the total time spent at the clinic was shorter in the intervention compared to usual care (median 34 vs. 51 minutes, *p* <0.001) (Table 3). The total time spent at the pharmacy including wait time was similar in the intervention and control clinics (median 8 vs. 8 minutes; *p* = 0.50), but clients had more direct contact time (i.e. excluding waiting) with the pharmacy staff in the direct‐to‐pharmacy intervention versus control clinics (median 7 vs. 4 minutes; *p* = 0.001). For HIV testing, the overall time spent on HIV testing including waiting was modestly shorter in the intervention but not statistically different compared to control clinics (median 21 vs. 25 minutes, *p* = 0.18). However, time directly spent on conducting the HIV test was unchanged for both groups (20 vs. 20 minutes; *p* = 0.50).

### 3.4 Completion and safety of client HIVST for PrEP services and HIV seroconversion

Overall, 98% and 94% of clients who were due for HIV testing at month 1 and month 3 in the direct‐to‐pharmacy intervention completed HIVST as expected (Table 4). Most clients chose to use blood‐based HIVST kits compared to oral‐based tests: 63% at month 1 and 60% at month 3. During the client self‐testing process, 81% sought assistance with self‐testing at the first visit to use the self‐test kit but only 30% required assistance at subsequent follow‐up visit. As per protocol, 97% (37/38) of clients returning at month 6 received provider‐conducted rapid HIV testing to cross‐validate HIV status. Overall, there was no documented HIV seroconversion among clients on PrEP returning for refills during the study.

**Table 4:** HIV self‐testing and kit choices at follow‐up visits

	Month 1 (first HIVST test visit)	Month 3 (second HIVST test visit)
	*n* = 118	*n* = 91
Client due for an HIV test	117 (99%)	64 (70%)
Client performed HIVST^a^	115 (98%)	60 (94%)
Choice of HIVST kit		
Oral‐based	43 (37%)	24 (40%)
Blood‐based	72 (63%)	36 (60%)
Asked for assistance with HIVST	93 (81%)	18 (30%)
Reasons for asking for assistance (among those asking for assistance)		
First time with HIVST	64 (69%)	2 (11%)
Needed help interpreting results	14 (15%)	1 (6%)
Needed help with test setup	15 (16%)	15 (83%)
Client asked to see a clinician	10 (9%)	10 (16%)

^a^ Clients (*n* = 2 at month 1 and *n* = 4 at month 3) who did not use HIVST as expected had a provider‐led HIV test.

### 3.5 Client experience and satisfaction with direct‐to‐pharmacy PrEP refill pathway

Overall, 158 clients receiving PrEP in the direct‐to‐pharmacy intervention completed the satisfaction survey (Table 5). Acceptability for direct‐to‐pharmacy intervention and HIVST for PrEP services was very high with ≥95% of respondents reporting high satisfaction with their overall clinic experience and that both the direct‐to‐pharmacy intervention and self‐testing were acceptable. Similarly, most clients had favourable satisfaction with the time spent at the clinics, including 96% reporting that they had adequate contact time with PrEP providers. However, about 20% reported some concern about the stigma of receiving PrEP in HIV clinics.

**Table 5:** Client acceptability and satisfaction with direct‐to‐pharmacy PrEP refill pathway

Characteristic	Implementation phase, *n* = 158
**Satisfaction with the overall experience**	
Better than previous times I came for PrEP	155 (98%)
Same as previous times I came for PrEP	2 (1.3%)
Worse than previous times	1 (0.6%)
**Satisfaction with the wait time**	
Very satisfied	142 (90%)
Somewhat satisfied	11 (7.0%)
Somewhat not satisfied	3 (1.9%)
Not satisfied at all	2 (1.3%)
**Feeling about wait time**	
Waited too much	21 (13%)
Waited just the right amount	109 (69%)
Waited too little	28 (18%)
**Had adequate time with PrEP providers**	
Yes	152 (96%)
No	6 (3.8%)
**Acceptability of direct‐to‐pharmacy PrEP**	
Very acceptable	154 (97%)
Equally acceptable	2 (1.3%)
Less acceptable	1 (0.6%)
Not acceptable at all	1 (0.6%)
**Acceptability of HIV self‐test**	
Very acceptable	143 (94%)
Equally acceptable	5 (3.3%)
Less acceptable	4 (2.6%)
**Concern about stigma**	
Very concerned	10 (6.3%)
Somewhat concerned	24 (15%)
Somewhat not concerned	15 (9.5%)
Not concerned at all	109 (69%)
**Likelihood of continuing PrEP services at this clinic**	
Very likely to continue	154 (97%)
Somewhat likely	2 (1.3%)
Not sure	2 (1.3%)

## 4 DISCUSSION

In this pilot implementation study, a client‐centred differentiated PrEP delivery approach with direct‐to‐pharmacy PrEP refill visits significantly reduced PrEP clinic visit time by more than one‐third and showed potential to improve continuation on PrEP in public health facilities in Kenya without diminishing adherence. Client HIVST at PrEP refill visits to support facility‐based oral PrEP provision was highly acceptable and safe in the context of effective oral PrEP use. The improvement in clinic efficiency and PrEP persistence demonstrates the potential for pharmacist‐led PrEP care to streamline facility‐based oral PrEP delivery and warrants further evaluation.

In comparison to HIV treatment, delivery of oral PrEP to healthy clients requires relatively fewer clinical components—HIV testing, adherence, risk reduction counselling, PrEP side effects, PrEP prescribing and dispensing. We have shown that all these can be done in a streamlined facility pharmacist‐led pathway in Kenyan public health facilities with demonstrable gains in clinic efficiency. Importantly, the direct‐to‐pharmacy pathway was highly acceptable among PrEP users. Furthermore, users with prior experience receiving PrEP in the usual clinic flow expressed overwhelmingly better clinic experience and higher levels of satisfaction with the direct‐to‐pharmacy refill visit compared to the usual clinic care. HIV stigma is an important barrier to PrEP access delivered in HIV care clinics [30]. This study was conducted in facility pharmacies dedicated to serving clients living with HIV and about 20% of PrEP clients expressed concerns about stigma. It is possible that implementing this model in pharmacies located in other facility departments such as the outpatient department or in community pharmacies may help to minimize the impact of stigma on PrEP access in facility‐delivery models.

In many low‐income countries, health facilities are often overcrowded, resulting in long wait times, and rushed medical care that may deter individuals from returning to the healthcare facility for follow‐up care. Our work and that of others have shown that young healthy individuals are less inclined to frequent healthcare facilities or overstay during visits for HIV preventive care [31] and several demonstration and implementation projects have consistently shown low PrEP persistence [32, 33, 34, 35]. The direct‐to‐pharmacy refill visits resulted in a significant gain in clinic efficiency by mostly cutting out redundant waiting time which could help minimize some of these barriers. One challenge associated with task‐shifting interventions is the potential for bottlenecks and overload to be transferred to a new service point. However, with existing staffing, this pharmacist‐led PrEP delivery intervention did not result in changes in the overall time spent by clients at the pharmacy. Instead, it provided more opportunities for increased interaction with the pharmacy staff, primarily driven by discussion and evaluation of HIV risk and ongoing need for PrEP.

HIV testing is a central component of PrEP delivery. In the current HIV testing protocols, HIV testing is conducted by providers. However, incorporating HIVST while waiting for other services has tremendous potential to streamline the delivery of oral PrEP, as it can free up the provider's workload and time to concentrate on other urgent services. We found that HIVST was safe and highly acceptable among PrEP clients and the need for help with using the self‐testing became less frequent during subsequent visits as clients became comfortable with using the kits and interpretation. Adherence to PrEP, as measured by TFV‐DP concentration in DBS was high and there was no documented HIV acquisition among clients on PrEP during the study period. These data suggest that, in the context of effective oral PrEP use, HIVST may be a safe HIV testing modality for facility‐based PrEP refill visits, as the risk of HIV acquisition is demonstrably diminished by the effective protection from PrEP use [2]. These data are particularly relevant in the context of real‐world oral PrEP delivery which does not reimburse clients for return visits as opposed to research studies, meaning client return for refills is a clear demonstration of their HIV prevention needs and motivation to use PrEP. We observed that clients spent about the same amount of time on self‐testing at the intervention clinics as the provider‐conducted HIV testing at the control clinics, but client HIVST resulted in clinic efficiency by freeing up about 20 minutes of the clinical provider time to perform other services.

We acknowledge that the study has several limitations. First, the study was not randomized, blinded or powered for cluster comparison. The direct‐to‐pharmacy intervention necessitated significant changes in usual clinic operations and needed to be studied initially at a few clinics before it could be tested in a more rigorous cluster randomized design. Thus, a quasi‐experimental design with control clinics provided a reasonable approach for this pilot intervention. Second, our study primarily relied on programme data abstracted from client records. This limitation meant that only variables available on MOH tools could be analysed, which limited our ability to assess important individual‐level variables. Third, the study population only represents individuals accessing PrEP in participating HIV care settings and may not be generalizable to other populations receiving PrEP outside of HIV care clinics. Fourth, we did not have qualitative data from providers and users which could have provided valuable insights, but planned qualitative analyses from this setting will help provide complementary contextual understanding. Fifth, COVID‐19 lockdowns occurred during the entire part of the pre‐implementation phase, while the intervention implementation started right after the relaxation of these measures. Regardless of the COVID‐19 pandemic impact, the overall observed PrEP persistence was somewhat low despite the significant clinic‐level efficiency gain from the direct‐to‐pharmacy intervention. This indicates that complementary non‐facility PrEP delivery models are needed to help clients who may have challenges frequenting traditional health facilities. Similarly, improving equitable access to emerging novel long‐acting PrEP options to improve product choices that align with user preferences and life circumstances may help to improve appropriate PrEP use. Beyond health system factors, individual, socio‐behaviour and structural factors that impact access to and utilization of prevention services and implementation strategies to address them also need to be clearly defined. Despite these limitations, our study demonstrated that a direct‐to‐pharmacy PrEP refill visit was feasible and highly acceptable and may have tremendous potential to streamline facility‐based care for oral PrEP delivery in this setting.

## 5 CONCLUSIONS

A client‐centred direct‐to‐pharmacy PrEP refill visit supported with client HIVST significantly reduced clinic visit time by more than a third and improved PrEP continuation in Kenya without diminishing adherence in public health HIV clinics in Kenya. As such, a differentiated facility‐based PrEP delivery with direct‐to‐pharmacy PrEP refill visits plus client HIVST has tremendous potential to streamline facility‐based care for oral PrEP.

## COMPETING INTERESTS

JMB is an employee of Gilead Sciences outside of this present work. KKM has received an Investigator Sponsored Research Grant from Gilead Sciences not related to this work awarded to the University of Washington. All other authors declare no competing interests.

## AUTHORS’ CONTRIBUTIONS

Study conceptualization and funding acquisition: KKM. Data collection tool development: KKM, KN, MM and GM. Data collection: KKM, KN, NRM, MM, DM, SM, LE, GM, VO and EO. Project administration: KKM, KN, NRM and JMB. Data analysis: KZ. Writing—original draft: KBZ and KKM. Writing—review and editing: All authors. All authors participated in the critical review and have read and approved the final manuscript.

## FUNDING

The study was supported by the National Institute of Mental Health of the US National Institutes of Health (grants R00MH118134 and R01MH123267).

## DISCLAIMER

The project funders had no role in the study design or writing of the report.

## Supporting information

Supplemental file1: PrEP Rapid Assessment Screening Tool

## Data Availability

Public sharing of individual participant data was not included in the informed consent form of the project and cannot be posted in a Supplementary File or a public repository because of legal and ethical restrictions. De‐identified data underlying this project can be made available to interested researchers upon reasonable request by contacting the International Clinical Research Center at the University of Washington (icrc@uw.edu).
