# Supplementary figures and images for: Effect of differentiated direct‐to‐pharmacy PrEP refill visits supported with client HIV self‐testing on clinic visit time and early PrEP continuation
Source: J Int AIDS Soc. 2024 Mar 6;27(3):e26222. doi: 10.1002/jia2.26222 (PMC10935714; doi:10.1002/jia2.26222)

Supplemental file1: PrEP Rapid Assessment Screening Tool.


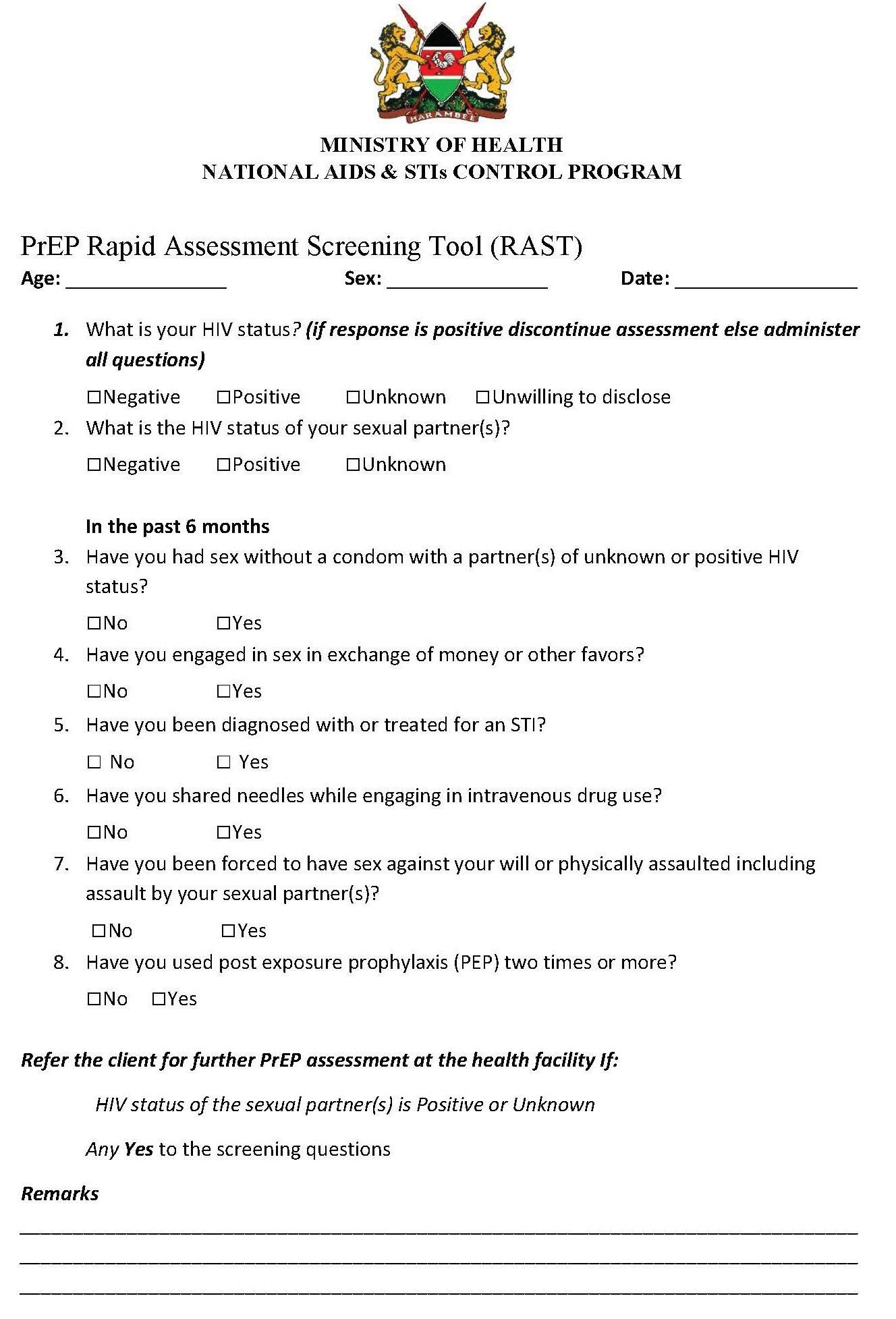

Supplement: Supplementary file 1 — Supplemental file1: PrEP Rapid Assessment Screening Tool [file JIA2-27-e26222-s001.docx]
